# Factors associated with treatment intensification in child and adolescent psychiatry: a cross-sectional study

**DOI:** 10.1186/s12888-018-1874-9

**Published:** 2018-09-10

**Authors:** Richard Vijverberg, Robert Ferdinand, Aartjan Beekman, Berno van Meijel

**Affiliations:** 1 Department of Child and Adolescent Psychiatry, GGZ Delfland, PO-box 5016, 2600 GA Delft, The Netherlands; 2 Amsterdam UMC, location VUmc and GGZ inGeest, Department of psychiatry, Amsterdam, The Netherlands; 3 Amsterdam Public Health Research Institute, Amsterdam, The Netherlands; 4 Inholland University of Applied Sciences, Amsterdam, The Netherlands; 5 GGZ-VS, Academy for Masters in Advanced Nursing Practice, Utrecht, The Netherlands; 6 Parnassia Psychiatric Institute, The Hague, The Netherlands

**Keywords:** Child and adolescent psychiatry, Assertive community treatment, Assertive outreach, Predictors, Risk factors

## Abstract

**Background:**

More knowledge about characteristics of children and adolescents who need intensive levels of psychiatric treatment is important to improve treatment approaches. These characteristics were investigated in those who need youth Assertive Community Treatment (youth-ACT).

**Method:**

A cross-sectional study among children/adolescents and their parents treated in either a regular outpatient clinic or a youth-ACT setting in a specialized mental health treatment center in the Netherlands.

**Results:**

Child, parent and family/social context factors were associated with treatment intensification from regular outpatient care to youth-ACT. The combination of the child, parent, and family/social context factors adds substantially to the predictive power of the model (Nagelkerke R^2^ increasing from 36 to 45% for the three domains separately, to 61% when all domains are combined). The strongest predictors are the severity of psychiatric disorders of the child, parental stress, and domestic violence.

**Conclusions:**

Using a wide variety of variables that are potentially associated with treatment intensification from regular outpatient clinic to youth-ACT, we constructed a regression model illustrating a relatively strong relation between the predictor variables and the outcome (Nagelkerke R^2^ = 0.61), with three strong predictors, i.e. severity of psychiatric disorders of the child, parental stress, and domestic violence. This emphasizes the importance of a system-oriented approach with primary attention for problem solving and stress reduction within the system, in addition to the psychiatric treatment of the child, and possibly also the parents.

## Background

Ten to 20 % of the children and adolescents in the general population suffer from a psychiatric disorder [1–3]. With the general practitioner as the gate keeper, most of the Dutch children and adolescents with psychiatric disorders are referred to outpatient clinics [4, 5]. If more intensive mental health care is necessary, children or adolescents can be referred by the general practitioner or via the outpatient clinic to youth Assertive Community Treatment (youth-ACT). This is an intensive home-based treatment that is provided by a multidisciplinary team of mental health care professionals who have small caseloads (size< 15).

Existing studies (only four) mainly studied child factors (and not: variables pertaining to parents) [6, 7], only pertained to children (and not to adolescents) [7], used small samples [8], or only studied children with autism spectrum disorder [9]. Two of the four studies were conducted more than 20 years ago [6, 7]. To increase scientific knowledge regarding the intensification of outpatient psychiatric treatment in children and adolescents we (1) studied a larger sample, and (2) examined child, parent, and family/social context factors that might predict intensification of outpatient treatment, in (3) both children and adolescents.

More knowledge about factors associated with intensifying treatment from regular outpatient care into youth-ACT is important from the perspective of prevention because it offers opportunities to determine which factors should be targeted with treatment to prevent increase in psychopathology and deterioration of functioning, ultimately leading to referral to a more intensive form of mental health care [6–9]. By identifying factors associated with intensifying treatment, mental health care professionals are encouraged to determine at an early stage whether regular outpatient care can be expected to be effective or if they should consider treatment intensification [9]. More precision in the allocation of care for those who need intensive treatment may help avoid exposure of patients to treatments that will prove to be ineffective, and lead to unnecessary delay in recovery [10, 11]. Conversely, in the vast majority of children and adolescents a more intensive form of treatment than outpatient care is not necessary, so referral to a setting such as youth-ACT would be inefficient for most patients [12–17].

The aim of this study was to investigate factors that are associated with treatment intensification from regular outpatient care into youth-ACT. We aimed to include variables on the child, parent and family/social context levels which are known from the literature to be associated with mental health of children [6–9, 18].

Our a priori hypotheses were that children and adolescents in whom outpatient treatment is intensified into youth-ACT have significantly more severe psychiatric disorders, more care needs, lower quality of life, and an older age [6–9]. Further, we expected that parents of children and adolescents in whom outpatient treatment is intensified into youth-ACT have higher levels of parental psychiatric disorders, more care needs, lower quality of life, higher levels of parental stress, and a poorer parental ability to deal adequately with the psychiatric problems of the child. Studies that link functioning of parents with the utilization of inpatient care of children and adolescents support these hypotheses [7–9]. At the family/social context level, we expected that treatment intensification from regular outpatient care into youth-ACT is associated with a parent being single parent, a larger number of children in the family, more domestic violence, more financial problems, less social support, and low family socioeconomic status (SES) [7–9].

## Method

### Design

We conducted a cross-sectional study with children/adolescents and their parents who were treated in either a regular outpatient clinic or a youth-ACT setting.

### Setting

The study was carried out between September 2014 and July 2016 in a specialized treatment center for psychiatric disorders in the Netherlands, GGZ Delfland. Two outpatient clinics and one youth-ACT team, who served patients in the same geographical area, were included. The two outpatient clinics carry out diagnostic assessments and treatment of children/adolescents using a multidisciplinary team. Each team consists of one child psychiatrist, six psychologists, and one nurse practitioner.

The youth-ACT team provides treatment based on the following elements and principles: (a) home-based multidisciplinary treatment, (b) intensity of treatment is scaled up or down according to the severity of current psychiatric symptoms and level of functioning of the patient, (c) small caseloads (size< 15), (d) focused on patients who are difficult to reach, (e) case management, (f) early intervention, (g) family support, (h) reintegration/vocational and educational therapy, (i) medication when appropriate. The youth-ACT team consists of one child psychiatrist, five psychologists, three nurse practitioners and two psychiatric nurses.

### Participants

**Fig. 1:**
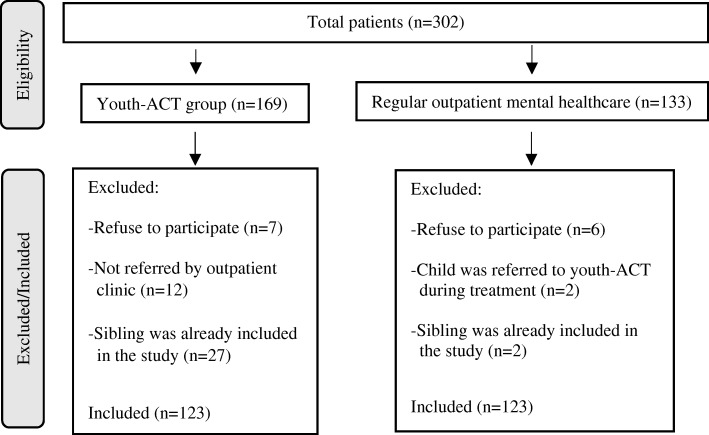
Participant flow diagram

Figure 1 presents the flowchart of the inclusion process. To be included, participants in both treatment settings had to meet the following inclusion criteria: (a) children/adolescents aged between 4 and 18 years; (b) with a DSM-IV diagnosis; and (c) had a parent who fulfilled the role of primary caregiver. Because the involvement of parents in raising a child can vary widely [19], especially when it concerns single parent families [20], only the parent who fulfilled the role of primary caregiver was included in this study. Only children who were referred from an outpatient clinic, were included in the youth-ACT sample.

Included outpatients who were later referred to youth-ACT were excluded from the outpatient sample and included in the youth-ACT sample. Also, children were excluded when a sibling or other child living in the same household already participated in the study.

### Ethical approval

The study was approved by the Medical Ethics Committee on Research Involving Human Subjects of the VU University Medical Centre, Amsterdam (protocol no. 2015.245). Participants received written and oral information, separately for children and parents, about the study and were included after giving informed consent.

### Measurement instruments

#### Child factors

For the assessment of psychiatric diagnoses, we used the Neuropsychiatric Interview for Children and Adolescent (MINI-KID), supplemented with clinical diagnoses that were not included in the MINI-KID [21].

The Health of the Nation Outcomes Scales Child and Adolescents Mental Health (HoNOSCA) was used to assess the severity of psychiatric disorders [22]. The HoNOSCA covers 15 items to be scored on a 5-point severity scale ranging from `no problems’ (0) to `severe problems’ (4). To calculate HoNOSCA-sum score, we used the items 1 to 13, since items 14 and 15 do not provide information about the mental health situation of the child.

The Camberwell Assessment of Need Short Appraisal Schedule (CANSAS) was used to assess the care needs of the child [23]. The CANSAS covers 25 care need items which are scored on a nominal 3-point scale ‘no need’ (1), `met need’ (2), and ‘unmet need’ (3). To calculate the CANSAS-sum score, the sum of met and unmet needs of the 25 items was computed.

The Kidscreen-27 was used to assess the health-related quality of life of children [24]. The Kidscreen is a self-report questionnaire that consists of 27 items to be scored on a 5-point scale ranging from ‘never’ (0) to ‘always’ (4). The Kidscreen sum score was calculated by adding up the scores of the 27 items.

A designated client-based standardized questionnaire (DEMOG) was used to measure the following demographic characteristics of the child: (a) age, (b) gender, and (c) country of birth [25]. This is a standardized client-based questionnaire to measure demographic characteristics.

#### Parental factors

The Health of the Nation Outcomes Scales (HoNOS) was used to assess the severity of psychiatric disorders of the parent [26–28]. The HoNOS covers 12 items to be scored on a 5-point severity scale ranging from `no problems’ (0) to `severe problems’ (4). The HoNOS sum score was calculated by adding up the scores of the 12 items.

Item 14 of the HoNOSCA was used to assess the parental knowledge about the difficulties of the child from the perception of the parent.

As with the children, the CANSAS was used by parents as a self-report scale to assess their care needs.

Parenting stress was measured using a Parenting Stress Scale. The primary caregivers rated their level of parenting stress on a scale ranging from 1 to 10.

The Manchester Short Assessment of quality of life (MANSA-16) was used to assess the health-related quality of life of the parent [29, 30]. The MANSA consists of 16 items to be scored on a 7-point scale ranging from `could not be worse’ (1) to `could not be better’ (7). The MANSA-16 sum score was calculated by adding up the scores of the 16 items.

DEMOG-adult was used to measure the following demographic characteristics of the parent: (a) age, (b) gender, and (c) country of birth [25].

#### Family/social context factors

The DEMOG-Adult was also used to assess living situation, family composition, and socio-economic status (SES; expressed in educational achievements and ethnic background).

Domestic violence was registered by a self-developed form to be scored ‘yes’ or ‘no’. It was assessed whether the client, parents, and siblings had used violence against other family members in the present.

Items 3 and 6 of the MANSA were used separately to assess social support and financial problems of the family, respectively.

### Data-analysis

Analyses were performed using SPSS 24.0. The descriptive statistics of the outpatient sample and youth-ACT sample were calculated on item or scale level. Next, we conducted three series of univariable logistic regression analyses, with treatment setting as dependent variable, to identify candidate predictors for the multivariable regression models. The first series concerned candidate predictors at the level of the child: severity of psychiatric disorders, care needs, quality of life, and age. The second series concerned candidate predictors at the level of the parent: severity of psychiatric disorders of the parent, care needs, quality of life, parental stress, and (lack of) knowledge pertaining to difficulties of the child. The third series concerned candidate predictors at the level of family/social context: living situation, the number of children, financial problems, educational achievements and ethnic background of the primary caregiver, domestic violence, social support and financial problems.

By using this step-by-step approach, we created multivariable regression models that did not violate the statistical rule of 10 events per 1 variable and ensured the validity of the analysis [31, 32]. First, for each level (child, parent, and family/social context) we conducted three separate multivariable analyses in which we entered the predictors that were (borderline)-significant (*P* < 0.10) in the univariable analyses. Finally, we conducted a stepwise multivariable analyses investigating the three levels (child, parent, and family/social context) together.

The assumptions of the logistic regression analyses (multicollinearity) were tested for indications of multicollinearity by examining the variance inflation factor (VIF) and tolerance values. No violations of limits were found (VIF range 1.00–2.00; tolerance between 0.45 and 1.00), indicating that there was no indication of multicollinearity [33]. Further, the assumptions of linearity and homoscedasticity were tested by creating a scatter plot of the standardized residuals. The distribution of the residuals was reasonably rectangular, and most of the scores were in the centre. Thus, the assumption of linearity and homoscedasticity were met in this study [33, 34]. The Hosmer and Lemeshow goodness-of-fit test was used to measure the predictive value of the model [33]. To obtain an overall indication of how well the models performed, we used the Omnibus test [33]. The Nagelkerke R^2^ was used to provide an indication of the strength of the relation between the predictor variables and the outcome variable [33]. The discrimination accuracy of the model was examined by using the area under the curve (AUC) of the receiver operating characteristic curve (ROC curve) and were categorized as fail (0.50–0.60); poor (0.60–0.70); fair (0.70–0.80); good (0.80–0.90); or excellent (0.90–1.00) [35].

## Results

### Characteristics of the sample

Characteristics of the study sample (*n* = 246) are presented in Tables 1 and 2. The outpatient sample (*n* = 123) comprised 56 girls (45.5%) and 67 boys (54.5%) with an average age of 11.8 years. The most frequent clinical diagnoses were attention deficit hyperactivity disorder (43.1%), anxiety disorder (31.7%), behavioral disorder (12.0%), or mood disorder (6.5%). In the outpatient sample, most primary care givers (*n* = 123) were mothers (99.2%) with an average age of 41.0 years. The majority of mothers (66.6%) had a paid job and 73.8% of these mothers raised their children in a two-parent household.

**Table 1:** Sample characteristics of the child or adolescent who received treatment

	Outpatient	Youth-ACT
	Child (*n*^1^=123)	Child (*n* = 123)
Age (sd^2^)	Total mean 11.8 (3.2)	Total mean 13.0 (3.2)
	range 6–17	range 4–18
	Girls mean 13.0 (3.4)	Girls mean 13.7 (3.0)
	range 6–17	range 4–18
	Boys mean 11.1 (2.9)	Boys mean 12.5 (3.3)
	range 6–17	range 6–17
Gender	Girls 45.5%	Girls 42.3%
	Boys 54.5%	Boys 57.7%
Country of birth	Holland 96.7%	Holland 95.1%
	Other 3.3%	Other 4.9%
Clinical diagnoses	Mood 6.5%	Mood 37.4%
	Anxiety 31.7%	Anxiety 41.5%
	Behavior 12.0%	Behavior 30.0%
	Psychotic 0.0%	Psychotic 4.0%
	ASD^3^11.4%	ASD 40.7%
	ADHD^4^ 43.1%	ADHD 42.3%
	Somatoform 0.8%	Somatoform 13.8%
	Drugs/alcohol 0.0%	Drugs/alcohol 3.2%
	Mental retard 3.2%	Mental retard 8.1%
	Personality 0.0%	Personality 5.7%
	Other 0.8%	Other 3.2%
GAF^5^-score (sd)	Mean 55.0 (5.4)	Mean 45.7 (8.1)
	Range 45–75	Range 15–60
Living situation	Single parent 26.2%	Single parent 42.1%
	Two parent 73.8%	Two parent 57.9%

^1^*n* number of included patients

^2^*sd* standard deviation

^3^*ASD* Autism spectrum disorder

^4^*ADHD* Attention deficit hyperactivity disorder

^5^*GAF* General assessment of functioning

**Table 2:** Sample characteristics of the parent who fulfilled the role of primary caregiver

	Outpatient	Youth-ACT
	Parent^1^ (*n*^2^=123)	Parent (*n* = 123)
Age (sd^3^)	Total mean 41.0 (6.2)	Total mean 43.7 (7.3)
	range 27–55	range 24–70
Status	Mother 99.2%	Mother 98.4%
	Father 0.8%	Father 1.6%
Country of birth	Holland 78.0%	Holland 88.6%
	Other 22.0%	Other 11.4%
Education status	Basic 15.4%	Basic 25.3%
	Intermediate 22.0%	Intermediate 29.2%
	High 62.6%	High 45.5%
Employment status	Paid job 66.6%	Paid job 35.8%
	No paid job 33.4%	No paid job 64.2%

^1^*Parent* primary care giver

^2^*n* number of included patients

^3^*sd* standard deviation

The youth-ACT sample (*n* = 123) comprised 52 girls (42.3%) and boys 71 (57.7%) with an average age of 13.0 years. The most frequent clinical diagnoses were attention deficit hyperactivity disorder (42.3%), anxiety disorder (41.5%), behavioral disorder (30.0%), and mood disorder (37.0%). In the youth-ACT sample 98.4% of the primary caregivers (*n* = 123) were mothers with an average age of 43.7 years. The majority of mothers (64.2%) did not have a paid job and 57.9% of these mothers raised their children in a two-parent household.

### Predictors youth-ACT

#### Univariable analyses

As presented in Table 3, the univariable analyses shows that treatment intensification from outpatient care into youth-ACT was predicted by all variables, with the exception of educational status of the primary caregiver (*P* = 0.210, *P* = 0.312), social support (*P* = 0.118), and number of children in the household (*P* = 0.965).

**Table 3:** Predictors of youth-ACT

Level of predictors	Univariable model^1^			Multivariable model^2^		
	n^3^	OR^4^ (95% CI^5^)	*P*-value	n	OR (95% CI)	*P*-value^6^
Child^8^				225		
HoNOSCA^7^	246	1.29 (1.21–1.38)	<.001		1.27 (1.18–1.37)	<.001
CANSAS^7^	243	1.06 (1.00–1.12)	0.034		0.93 (0.87–1.01)	0.051
Kidscreen^7^	228	0.94 (0.92–0.97)	<.001		0.97 (0.95–1.00)	0.213
Age	246					
4–11 years old	93					
12–18 years old	153	2.24 (1.32–3.80)	0.003		1.41 (0.71–2.81)	0.322
Parent^9^				238		
HoNOS^7^	244	1.22 (1.15–1.30)	<.001		1.22 (1.10–1.35)	<.001
CANSAS^7^	246	1.12 (1.07–1.12)	<.001		0.99 (0.92–1.06)	0.802
MANSA^7^	240	0.93 (0.91–0.96)	<.001		1.01 (0.97–1.06)	0.568
Parental stress	245	1.53 (1.34–1.76)	<.001		1.42 (1.21–1.67)	0.002
Lack of knowledge pertaining to difficulties	246	1.66 (1.30–2.13)	<.001		1.60 (1.19–2.15)	0.003
Family and social context^10^				243		
Living situation	243					
*Two parents*	160					
*Single parent*	83	2.05 (1.19–3.42)	0.009		1.30 (0.67–2.55)	0.437
Domestic violence	245	12.05 (6.20–23.42)	<.001		11.27 (5.56–22.86)	<.001
Financial problems	243	1.32 (1.11–1.56)	0.015		1.06 (0.86–1.31)	0.593
Ethnic background primary caregiver	246					
*Dutch*	236					
*Other*	10	0.46 (0.23–0.92)	0.029		0.40 (0.18–0.91)	0.028
Social support	243	0.52 (0.23–1.18)	0.118			
Educational status	246					
*Basic*	39					
*Intermediate*	74	1.65 (0.75–3.63)	0.210			
*High*	133	0.69 (0.34–1.41)	0.312			
Number of children	245	0.99 (0.76–1.30)	0.965			

^1^Univariable: binary logistic analyses of each candidate predictor preformed separately

^2^Multivariable: binary logistic regression analysis of the predictors that were significant in the univariable analysis, performed simultaneously

^3^*n* number of patients

^4^*OR* Odds Ratio

^5^*CI* Confidence interval

^6^*P*-value< 0.10 is considered statistically significant

^7^Sum-score

^8^Child-level: Omnibus test, Step *P* = 0.00, Model *P* = < 0.00, Hosmer-Lemeshow, *P* = 0.68, Nagelkerke R^2^=0.43, AUC = 0.84, 95% CI 0.78–0.89, *P* < 0.001

^9^Parent-level: Omnibus test, Step *P* = 0.00, Model *P* = < 0.00, Hosmer-Lemeshow, *P* = 0.91, Nagelkerke R^2^=0.45, AUC = 0.85, 95% CI 0.80–0.89, *P* < 0.001

^10^Family-social context-level: Omnibus test, Step *P* = 0.00, Model *P* = < 0.00, Hosmer-Lemeshow, *P* = 0.58, Nagelkerke R^2^ = 0.36, AUC = 0.78, 95% CI 0.72–0.84, *P* < 0.001

At the child level, the referral to youth-ACT was predicted by the severity of the psychiatric disorders, assessed with the HoNOSCA (OR = 1.29, 95% CI 1.21–1.38, *P* < 0.001), the child’s care needs (OR = 1.06, 95% CI 1.00–1.12, *P* = 0.034), quality of life (OR = 0.94, 95% CI 0.92–0.97, *P* < 0.001), and age (OR = 2.24, 95% CI 1.32–3.80, *P* = 0.003).

At the parent level, the severity of psychiatric disorders of the parent predicted the referral to youth-ACT (OR = 1.22, 95% CI 1.15–1.30, *P* < 0.001), as did the parents’ care needs (OR = 1.12, 95% CI 1.07–112, *P* < 0.001), and the parents’ quality of life (OR = 0.93, 95% CI 0.91–0.96, *P* < 0.001). Also, the parental knowledge about the difficulties of the child and the perceived parental stress were significant predictors (respectively OR = 1.53 (95% CI 1.34–1.76, *P* < 0.001) and OR = 1.66 (95% CI 1.30–2.13, *P* < 0.001)).

At the family/social context level, being a single parent (OR = 2.05, 95% CI 1.19–3.42, *P* = 0.009), the presence of domestic violence (OR = 12.05, 95% CI 6.20–23.42, *P* < 0.001), having financial problems (OR = 1.32, 95% CI 1.11–1.56, *P* = 0.015), and ethnic background of the primary caregiver (OR = 0.46, 95% CI 0.23–0.92, *P* = 0.029) were significant predictors.

#### Multivariable analyses for each level separately

Table 3 shows that at the child level, quality of life (*P* = 0.213), and age of the child (*P* = 0.322) did not remain significant in the multivariable analysis. The child model showed a good fit of the data (Hosmer-Lemeshow, *P* = 0.681) and illustrated a relatively strong relation between the predictor variables and the outcome (Nagelkerke R^2^ = 0.43). The AUC under the ROC curve suggested that the model has a good classification ability to discriminate the referral to regular outpatient care with youth-ACT (AUC = 0.84, 95% CI 0.78–0.89, *P* < 0.001).

At the parent level, the care needs (*P* = 0.802) and quality of life (*P* = 0.568) did not remain significant. The model showed a good fit of the data (Hosmer-Lemeshow, *P* = 0.912) and illustrated a relatively strong relation between the predictor variables and the outcome (Nagelkerke R^2^ = 0.45). The model has a good classification ability to discriminate the referral to regular outpatient care with youth-ACT (AUC = 0.85, 95% CI 0.80–0.89, *P* < 0.001).

At the family/social context level, being a single parent (*P* = 0.437) and having financial problems (*P* = 0.593) did not remain significant in the multivariable analysis. The model fitted the data (Hosmer-Lemeshow, *P* = 0.576) and illustrated a relatively strong relation between the predictor variables and the outcome (Nagelkerke R^2^ = 0.36). The model has a fair classification ability to discriminate the referral to regular outpatient care with youth-ACT (AUC = 0.78, 95% CI 0.72–0.84, *P* < 0.001).

#### Multivariable analyses for all levels simultaneously

First, in the final logistic model, the severity of the psychiatric disorder of the child (OR = 1.31, 95% CI 1.22–1.41, *P* < 0.001) remained a significant predictor regarding referral to youth-ACT (see Table 4). However, the child’s care needs did not remain significant (*P* = 0.186). Second, the significant child and parent predictors together showed that all predictors remained significant: the severity of the psychiatric disorder of the child (OR = 1.21, 95% CI 1.12–1.30, *P* < 0.001) and parent (OR = 1.13, 95% CI 1.05–1.21, *P* < 0.001), parental stress (OR = 1.36, 95% CI 1.15–1.60, *P* < 0.001) and parental knowledge about the difficulties of the child (OR = 1.39, 95% CI 1.00–1.92, *P* = 0.049). Third, when the family/social context predictors were added, the severity of the psychiatric disorder of the child (OR = 1.19, 95% CI 1.11–1.29, *P* < 0.001), parental stress (OR = 1.35, 95% CI 1.13–1.62, *P* = 0.001), and domestic violence (OR = 5.19, 95% CI 2.20–12.26, *P* < 0.001) remained significant predictors. The severity of the psychiatric disorder of the parent (*P* = 0.085), parental knowledge about the difficulties of the child (*P* = 0.081) and ethnic background of the primary caregiver (*P* = 0.104) were no longer significantly associated with the dependent variable.

**Table 4:** Predictors of youth-ACT

Level of predictors	Child^1^		Child and parent^2^		Child, parent and family/social context^3^	
	Multivariable model^4^		Multivariable model		Multivariable model	
	OR^5^(95% CI^6^)	*P* ^*7*^	OR (95% CI)	*P*	OR (95% CI)	*P*
Child						
HoNOSCA^8^	1.31 (1.22–1.41)	<.001	1.21 (1.12–1.30)	<.001	1.19 (1.11–1.29)	<.001
CANSAS ^8^	0.96 (0.90–1.02)	0.186				
Parent^9^						
HoNOS^8^			1.13 (1.05–1.21)	<.001	1.07 (0.99–1.15)	0.085
Parental stress			1.36 (1.15–1.60)	<.001	1.35 (1.13–1.62)	0.001
Lack of knowledge pertaining to difficulties			1.39 (1.00–1.92)	0.049	1.34 (0.96–1.87)	0.081
Family/social context						
Domestic violence					5.19 (2.20–12.26)	<.001
Ethnic background primary caregiver						
*Dutch*						
*Other*					0.40 (0.13–1.21)	0.104

^1^Child-level: Omnibus test, Step *P* = 0.00, Model *P* = < 0.00, Hosmer-Lemeshow, *P* = 0.26, Nagelkerke R^2^=0.43, AUC = 0.84, 95% CI 0.78–0.89, *P* < 0.001

^2^Child-parent-level: Omnibus test, Step *P* = 0.00, Model *P* = < 0.00, Hosmer-Lemeshow, *P* = 0.17, Nagelkerke R^2^=0.56, AUC = 0.89, 95% CI 0.85–0.93, *P* < 0.001

^3^Child-parent-family/social context-level: Omnibus test, Step *P* = 0.00, Model *P* = < 0.00, Hosmer-Lemeshow, *P* = 0.51, Nagelkerke R^2^=0.61, AUC = 0.91, 95% CI 0.87–0.95, *P* < 0.001

^4^Multivariable: binary logistic regression analysis of all predictors entered simultaneously

^5^*OR* Odds Ratio

^6^*CI* Confidence interval

^7^*P*-value< 0.10 is considered statistically significant

^8^Sum-score

^9^Parent: primary caregiver

The model of the child, parent and family/social context predictors together showed a good fit of the data (Hosmer-Lemeshow, *P* = 0.511), and illustrated a relatively strong relation between the predictor variables and the outcome (Nagelkerke R^2^ = 0.61). The AUC under the ROC curve suggested that the model has an excellent classification ability to discriminate the referral to regular outpatient care with youth-ACT (AUC = 0.91, 95% CI 0.87–0.95, *P* < 0.001).

## Discussion

This cross-sectional study examined the patient, family and contextual variables that are associated with treatment intensification from regular outpatient care to a more intensive form of treatment: youth-ACT. To our knowledge, this is the first study that provides a much more detailed insight into the variables that are associated with intensifying outpatient treatment towards more intensive treatment. Through the step-by-step logical regression, a view is obtained on the hierarchy of these variables. By applying the step-by-step logistic regressions, we determined which variables are the strongest predictors of intensification of treatment. As hypothesized, we found many univariable associations between candidate predictors and intensification of treatment (see Table 3). However, not all variables that were entered as possible predictors of intensification of treatment were significant (see Table 3). In contrast to our expectations, significant effects of level of social support and educational status of parents on treatment intensification were not found. Number of children in the family did not predict treatment intensification as well.

Our findings indicate that children in whom outpatient care is intensified are likely to have parents with more psychiatric problems (see Table 3). A cross-sectional relation like this can be explained in three different ways: X caused Y, Y caused X or there is a third variable causing X and Y [36]. Hence, a possible explanation is that severe psychiatric problems of the child negatively affect a parent’s mental health [37]. Conversely, severe psychiatric problems in parents may also negatively affect a child’s mental health [37, 38]. Finally, third variables, for instance, similar genes, or living in a similar adverse environment, may influence mental health in parents and their children.

We also found that parents of children in whom outpatient care is intensified display high levels of parental distress. This association may indicate that serious psychiatric symptoms in the child, that require more intense treatment, cause a high level of distress in parents, or vice versa. But, a variable influencing both psychiatric symptoms in a child, as well as parental distress, may be present as well.

A relation was also found between treatment intensification and lower quality of life and more care needs in parents (see Table 3). Regardless of the direction or exact nature of associations between, on one hand, treatment intensification, and on the other hand, parental mental health, parental distress, parental quality of life, and parental care needs, it is clear that all associations found possibly indicate a diminished ability of the parent to support children who are at risk for treatment intensification. In other words, several parental characteristics that were found may negatively influence treatment effects in their children [39–42].

Existing guidelines for intensive forms of treatment suggest that children who are living in families where children and parents experience many problems need a system-oriented approach [43, 44]. However, a classical system-oriented approach does not seem to be sufficient, because this approach does not specifically focus on the psychiatric problems of parents, and on their care needs and quality of life. Our findings suggest that this may be necessary, in addition to the classic system-oriented approach.

When conducting multivariable analyses including all levels, three variables - severity of psychiatric disorder of the child, parental stress and domestic violence - remained significant. The logistic regression model that included these three predictors showed a strong relation between the predictor variables and the outcome illustrated by the Nagelkerke R^2^ of 0.61 and has an excellent ability to discriminate (AUC = 0.91, 95% CI 0.87–0.95, *P* < 0.001), indicating a high predictive value. We may conclude that children with severe psychiatric disorders, who live in a context where parents experience high levels of parental stress and where domestic violence takes place, are most likely to be referred from outpatient care to a more intensive form of treatment. A possible explanation is that there are negative reciprocal interactions patterns between domestic violence, parental stress and the severe psychiatric disorders in children [45]. When health care providers, together with patient and family members, are not able to break through these negative reciprocal interaction patterns when offering regular outpatient care, referral to more intensive treatment (such as youth ACT) is needed.

The effect sizes found in relation to domestic violence and parental stress are remarkable when we consider that existing guidelines for children and adolescents do not contain recommendations regarding stopping domestic violence and reducing parental stress [46–49]. Our results show that it is important to encourage guidelines to include recommendations regarding these issues.

For clinical practice, our findings indicate that health care professionals should pay extra attention to children with severe psychiatric disorders, parents who are stressed, and families characterized by domestic violence. A first step would be to assess (all of these) problems systematically. In case of high HoNOSCA-scores, it is especially important to assess parental stress and domestic violence as well. If problems exist in these areas, it may be important to focus treatment not only on reducing psychiatric problems, but also on parental stress and domestic violence. Another, important finding (see Table 4), is the prediction of treatment intensification by parental stress, and not by HONOS-scores of the parent in the final analysis. Our finding, parental stress scores being very important, is of clinical significance, because it shows that, instead of screening parents for a broad range of psychiatric disorders, which is time consuming, one single question (parental stress was assessed on a visual analogue scale in our study) regarding parental stress is sufficient to screen for children/adolescents with a poor prognosis.

To our knowledge, regular outpatient treatment and youth-ACT programs do often not incorporate specific modules targeted at parental stress and at domestic violence [43]. The present study shows that, by adding such modules to outpatient treatment and youth-ACT programs might decrease the need for ACT. This is not to say that in the daily practice of an outpatient clinic or youth-ACT team, mental healthcare providers do not pay attention to reducing parental stress and domestic violence, but it is different to focus treatment specifically on these problems.

### Strengths and limitations

This study has several strengths. First, to our knowledge, this is the first study that examined the patient, family and contextual variables that are associated with referral to regular outpatient care or youth-ACT together. Second, a methodological strength of this study is that the youth-ACT sample consisted of patients that were referred directly from outpatient clinics in the same geographical catchment area, which assured a fair comparison of both groups. Third, the data was collected from a relatively large sample (*n* = 246) and had limited missing values (3%). The power of the analyses was sufficient to draw relatively firm conclusions about the associations between characteristics of patients, their families and living context on the one hand, and treatment intensity on the other hand. Fourth, in order to prevent bias, the data in both samples were collected during the intake-phase of both types of treatment. A clear limitation of the study is that data were collected in one youth-ACT team and two outpatient clinics from the same mental health organization. Therefore, the results of this study cannot be generalized without reservations [36]. However, it is worthwhile to note that this study is not about a specific treatment modality, but about the phenomenon of intensification of outpatient treatment. This intensification may occur in various forms, but always involves the intensification of treatment compared to regular outpatient care. Although the Dutch situation and/or treatment facilities are specific, they are also very similar to the international guideline-based care for children and adolescents. Therefore, generalisability of our findings seems not only limited to Dutch situation, despite the variation in practice across various countries.

### Recommendations

Domestic violence and parental stress are strong predictors of treatment intensification. Therefore, research is needed to determine whether the addition of special modules targeted at domestic violence and parental stress can actually prevent intensification of treatment, but also to improve effects of intensive treatment.

To date, studies examining youth-ACT mainly focussed on child-related factors, such as severity of psychiatric symptoms, general functioning, and duration and frequency of psychiatric hospital admissions [50]. In line with our research, it is important that future research regarding treatment intensification to youth-ACT includes variables at child, parent and family/social context level. The results of this study emphasize the importance of a system-oriented approach with primary attention for problem solving and stress reduction within the system, in addition to the psychiatric treatment of the child, and possibly also the parents.

## Conclusion

To summarize, child, parent and family-social context factors predict treatment intensification from outpatient care to youth-ACT. Although each domain has a unique and important contribution to make, and although variables across domains are correlated, the combination of the three domains adds substantially to the predictive power of the model (increasing from Nagelkerke R^2^ 0.36–0.45 in the three domains separately, to 0.61 when all domains were combined). Nagelkerke R^2^ of 0.61 for treatment allocation is a high predictive power.

The strongest predictors regarding treatment intensification from outpatient care to youth-ACT are the severity of psychiatric disorders of the child, parental stress and domestic violence. From the perspective of prevention and effectiveness it is important to examine whether influencing parent and family-social context factors affects the mental health situation of the child and its need for youth-ACT.

## Abbreviations

ACT: Assertive Community Treatment
ADHD: Attention Deficit Hyperactivity Disorder
ASD: Autism Spectrum Disorder
AUC: Area under the curve
CANSAS: Camberwell Assessment of Need Short Appraisal Schedule
CI: Confidence Interval
Fig.: Figure
GAF: Global Assessment of Functioning
HoNOS: Health of the Nation Outcome Scales
HoNOSCA: Health of the Nation Outcome Scales Child and Adolescents Mental Health
MANSA: Manchester Short Assessment of quality of life
MINI-KID: MINI International Neuropsychiatric Interview for Children and Adolescents
OR: Odds Ratio
SD: Standard Deviation
SPSS: Statistical Package for the Social Sciences
